# Protective Effect of Hydrogen-Rich Saline on Spinal Cord Damage in Rats

**DOI:** 10.3390/ph16040527

**Published:** 2023-04-01

**Authors:** Murat Kayabaş, Levent Şahin, Mustafa Makav, Duried Alwazeer, Levent Aras, Serdar Yiğit, Tyler W. LeBaron

**Affiliations:** 1 Department of Neurosurgery, Faculty of Medicine, Kafkas University, 36100 Kars, Türkiye; 2 Department of Emergency Medicine, Faculty of Medicine, Kafkas University, 36100 Kars, Türkiye; 3 Department of Physiology, Faculty of Veterinary, Kafkas University, 36040 Kars, Türkiye; 4 Department of Nutrition and Dietetics, Faculty of Health Sciences, Iğdır University, 76000 Iğdır, Türkiye; 5 Department of Histology and Embryology, Faculty of Medicine, Kafkas University, 36100 Kars, Türkiye; 6 Department of Kinesiology and Outdoor Recreation, Southern Utah University, Cedar City, UT 84720, USA; 7 Molecular Hydrogen Institute, Enoch, UT 84721, USA

**Keywords:** apoptosis, cytokines, hydrogen-rich saline, reactive oxygen species, spinal cord injury

## Abstract

The anti-inflammatory and anti-apoptotic effects of molecular hydrogen, delivered as hydrogen-rich saline (HRS), on spinal cord injury was investigated. Four-month-old male Sprague Dawley rats (*n* = 24) were classified into four groups: (1) control—laminectomy only at T7-T10; (2) spinal injury—dura left intact, Tator and Rivlin clip compression model applied to the spinal cord for 1 min, no treatment given; (3) HRS group—applied intraperitoneally (i.p.) for seven days; and (4) spinal injury—HRS administered i.p. for seven days after laminectomy at T7–T10 level, leaving the dura intact and applying the Tator and Rivlin clip compression model to the spinal cord for 1 min. Levels of interleukin-6 (IL-6) and tumor necrosis factor-alpha (TNF-α) were measured in blood taken at day seven from all groups, and hematoxylin–eosin (H & E) and terminal deoxynucleotidyl transferase dUTP nick-end labeling (TUNEL) were used to stain the tissue samples. IL-6 and TNF-α levels were significantly lower in the group treated with HRS following the spinal cord injury compared to the group whose spinal cord was damaged. A decrease in apoptosis was also observed. The anti-inflammatory and anti-apoptotic effect of IL-6 may be a clinically useful adjuvant therapy after spinal cord injury.

## 1. Introduction

Post-traumatic spinal cord injury is a common cause of severe physical disability. It greatly impairs an individual’s quality of life by affecting mobility, sexual functions, and bladder control [1,2]. Spinal cord injury can be defined as an injury resulting from an injury to the spinal cord that completely or incompletely compromises its main functions (motor, sensory, autonomic and reflex). Spinal cord injury remains an important cause of morbidity and mortality in modern society [3]. Several animal studies have reported an improvement in neurological function when administering a variety of therapeutic agents on spinal cord injury including antioxidants [4,5]. However, a permanent and effective universal treatment protocol has not been developed. 

Primary spinal cord damage is defined by the type and severity of the trauma. Surgical decompression provides only moderate or limited functional recovery [6]. Secondary injury develops as part of the sequela induced by hypoxia-activated microcirculatory changes, oxidative stress, ischemia, necrosis, apoptosis, and edema after primary injury [7]. It is believed that reactive oxygen species (ROS) such as the superoxide anion (O_2_^−^), hydrogen peroxide (H_2_O_2_), and hydroxyl radical (^•^OH), as well as reactive nitrogen species (RNS) such as nitric oxide (NO^•^) and peroxynitrite (ONOO^−^) play important roles in secondary injury [8]. ROS and RNS initiate the expression of inflammation-related adhesion molecules, cytokines, and apoptosis by stimulating their respective gene regions. ROS also cause cytotoxicity by inducing the peroxidation of lipids in the cell membrane, degradation of proteins, and damage to DNA [9]. The increased ROS, RNS and inflammatory cytokines further promote programmed cell death (e.g., apoptosis, necroptosis, ferroptosis, etc.) [10]. These pro-inflammatory cytokines that activate the inflammatory process include interleukin-6 (IL-6) and tumor necrosis factor-alpha (TNF-α). TNF-α also activates other transcription factors that induce the production of additional pro-inflammatory cytokines. After spinal cord injury, IL-6 causes gliosis, and the neurotoxic effects of TNF-α cause apoptosis. Neuronal tissues are particularly susceptible to oxidative damage due to their high abundance of polyunsaturated fatty acids. Therefore, studies have been conducted on antioxidant protocols for the treatment of spinal cord injury [11]. For example, the treatment of rats with spinal cord injury with a peroxynitrite scavenger (i.e., tempol), demonstrated protective effects, and pharmacologically confirmed that peroxynitrite is also involved in the pathophysiology of spinal cord injuries [12].

Molecular hydrogen (H_2_ gas) has recently been recognized as a novel therapeutic with anti-oxidant, anti-inflammatory, and anti-apoptotic protective effects [13]. Importantly, H_2_ has a high cellular bioavailability due it is small molecular mass/size and its non-ionic, non-polar chemical property [14]. This allows the H_2_ molecule to easily permeate lipid membranes and reach subcellular organelles via simple passive diffusion. Additionally, compared to conventional antioxidants, molecular hydrogen does not react with physiologically important ROS that are needed for intracellular communication, but only reduces toxic oxidants such as the hydroxyl radical and the pernicious peroxynitrite molecule [15]. Hydrogen therapy has been shown to have beneficial effects in many animal models [16] as well as in human clinical studies [17]. 

For example, Ono et al. (2011) conducted a clinical trial treating patients with edaravone, an approved medical drug used for the treatment of stroke, combined with hydrogen-saturated saline, and investigated the effects on patients with brainstem infarction [18]. Magnetic resonance imaging indicators of patients treated with hydrogen and edaravone were found to be better than those treated with edaravone alone. This indicated that hydrogen-rich saline (HRS) has neuroprotective effects, which was also demonstrated earlier. For example, in a mouse model, Cai et al. (2008) studied the effect of hydrogen therapy on hypoxia/ischemia-induced brain injury, which is the main cause of neuronal cell death [19]. Seven-day-old rat pups were exposed to hypoxia (8% oxygen at 37 °C) for 90 min. Next, the mice were placed in a chamber filled with 2% H_2_ for 30, 60 or 120 min, respectively. Twenty-four hours after this treatment, caspase-3 and caspase-12 activities in the cortex and hippocampus were evaluated, as well as brain damage, Nissl and deoxynucleotidyl transferase dUTP nick-end labeling (TUNEL) staining. Depending on the duration, H_2_ treatment significantly reduced the number of positive TUNEL cells and their caspase-3 and 12 activities [19]. The results of these and other studies demonstrate that H_2_ has the ability to confer protection in the brain through the inhibition of neuronal apoptosis. 

Deep hypothermic circulatory arrest (DHCA) is the process of stopping the blood flow to the brain in sensitive brain surgeries and has been applied since 1950. When this process is applied for a long time, significant oxidative stress, inflammatory stress, and apoptosis develop in the body. Shen et al. (2011) studied the effect of hydrogen-rich saline administration in DHCA-treated mice [20]. HRS application provided a significant reduction in the severity of DHCA-induced brain damage by a variety of mechanisms, including improvement in oxidative stress, down-regulation of inflammatory factors, and a reduction in apoptosis. 

Additionally, many animal and human experimental studies have been conducted on hydrogen in Alzheimer’s disease [21]. Hydrogen-rich saline prevented Aβ-induced neuroinflammation and oxidative stress, which may contribute to the amelioration of memory dysfunction in a mouse model [22]. Furthermore, several studies have applied molecular hydrogen therapy to a variety of spinal cord injuries. For example, hydrogen-rich saline protected against a rat model of contuse spinal cord injury when administered immediately, and at 24 h and 48 h after injury. HRS suppressed oxidative stress and apoptosis [9,13].

Although there are many methods to deliver molecular hydrogen (e.g., inhalation or ingestion of H_2_ dissolved in water), HRS represents a portable, easy-to-apply, and safe method to deliver the H_2_ molecule [23]. It may be a preferred method for use on some injuries due the ability to control the dose and target its administration [24]. However, there are only limited studies that have evaluated the effects of HRS on an experimental model of spinal cord injury. The present study investigated the neuroprotective, histological, and anti-inflammatory therapeutic efficacy of hydrogen-rich saline therapy for spinal cord injury in a rat model.

## 2. Results

**Figure 1:**
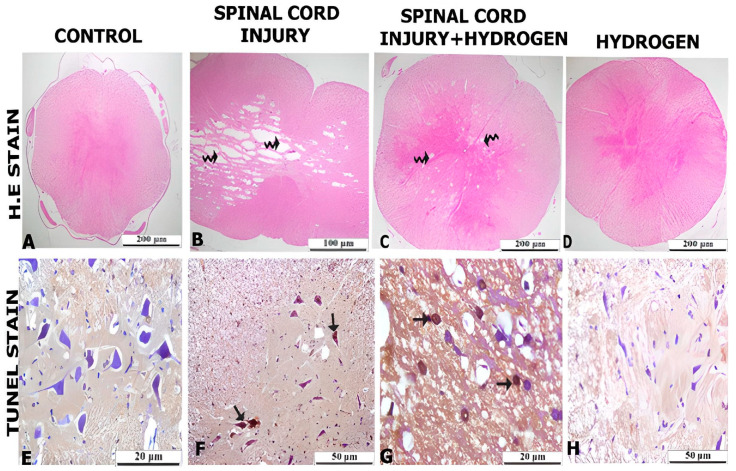
Histopathologic findings in spinal cord tissue. (**A**) Control group spinal cord H & E staining (bar: 200 µm). (**B**) Spinal cord injury group, curved arrow (bar: 100 µm). H & E staining of damaged lesion areas. (**C**) Spinal cord injury + hydrogen group, curved arrow: H & E staining of damaged lesion areas (bar: 200 µm). (**D**) Hydrogen group: H & E staining (bar: 200 µm). (**E**) Control group: terminal TUNEL staining (bar: 50 µm). (**F**) Spinal cord injury group: TUNEL staining, normal arrow, positive cells normal arrow gray matter: TUNEL-positive neurons in spinal cord–damaged group; TUNEL staining arrow (bar: 50 µm). (**G**) Spinal cord injury + hydrogen group: TUNEL staining (bar: 50 µm), normal arrow TUNEL-positive cells. (**H**) Hydrogen group: TUNEL staining (bar: 50 µm).

**Figure 2:**
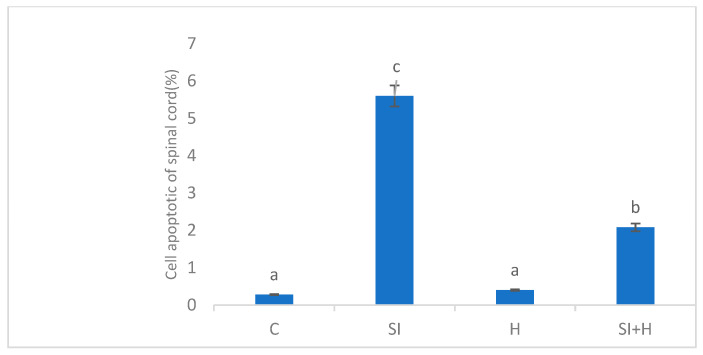
TUNEL staining representing cell apoptosis in the spinal cord of rats C: control, SI: Spinal cord injury, H: Hydrogen, SI + H: Spinal cord injury + hydrogen. There is no significant difference between groups denoted by the same letter (*p* > 0.05). There is a significant difference between the groups indicated with different letters (*p* ˂ 0.05).

The control group had a normal histological appearance according to hematoxylin–eosin (H & E) and TUNEL staining. H & E staining showed lesioned and local bleeding areas in the SI group, and TUNEL-positive staining of the neurons was observed. The lesioned areas in the SI-H group decreased compared to the SI group, while glia cells showed TUNEL-positive staining, and the neurons showed slightly positive staining. No abnormal structures were found in either the H & E and TUNEL staining in the SI-H group (Figure 1). The results of the Tukey’s test showed no significant difference between the C and H groups (*p* > 0.05); however, there was a significant difference between the C group and both the SI and SI-H groups (*p* ˂ 0.05). There was also a significant difference between the SI and the SI-H groups (*p* ˂ 0.05) (Figure 2).

**Figure 3:**
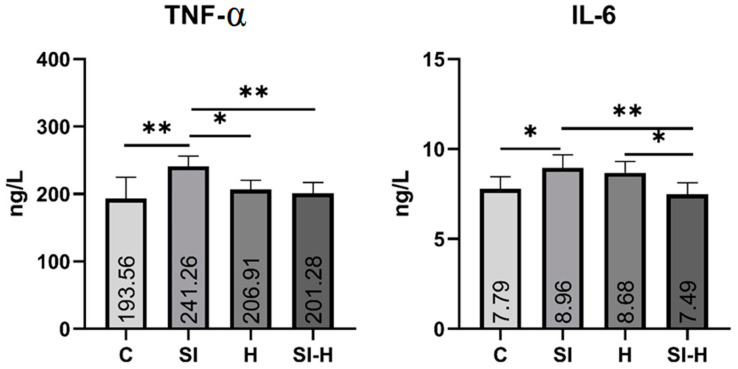
Comparison of molecular TNF-α and IL-6 between groups, Means and Std. Errors of the four groups for biochemical parameters. * *p* < 0.05, ** *p* < 0.01.

Blood IL-6 and TNF-α values were measured in intracardiac serum from rats in all groups as shown in Figure 3.

TNF-α and IL-6 values increased in each group compared to those in the C group. TNF-α and IL-6 values in the SI group were significantly higher than those in the C group (*p* < 0.05). The TNF-α and IL-6 values were also significantly lower between the SI-H and the SI groups (*p* < 0.05).

It was also found that TNF-α and IL-6 values decreased significantly in the SI-H group and reached values that did not differ statistically from those in the C group. When H and C group values were compared, no significant difference was found in TNF-α and IL-6 values (*p* > 0.05). Based on these results, the TNF-α and IL-6 levels in the serum increased after spinal cord injury, while they decreased in the SI-H group.

## 3. Discussion

Traumatic spinal cord injury has devastating consequences for the physical, social and occupational well-being of patients. The demographics of spinal cord injury are changing so much that an increasing proportion of older individuals are affected. Pathophysiologically, the initial mechanical trauma (primary injury) permeabilizes neurons and glia. This initiates a secondary injury cascade that leads to progressive cell death and spinal cord damage over the following weeks. Over time, the lesion remodels and forms cystic cavitations and a glial scar, both of which strongly inhibit regeneration. Various animal models and complementary behavioral tests of spinal cord injury have been developed to mimic this pathological process and lay the foundation for the development of preclinical and translational neuroprotective and neuro-regenerative strategies [25]. 

Several experimental spinal cord injury studies have investigated the therapeutic properties of different agents that have neuroprotective effects on secondary injury [26,27]. Some of the agents used have been steroids, antioxidants/free radical scavengers, opiate-receptor antagonists, gangliosides, thyrotropin-releasing hormone and its analogues, glutamate receptor blockers, arachidonic acid modulators, calcium-channel antagonists, sodium-channel blockers, monoamine modulators, immunosuppressants, nonsteroidal anti-inflammatories, and growth factors. Some studies have shown partial improvements in neurological functions using these types of therapeutics [28,29]. However, there is no single therapeutic that is overtly clinically effective, and many therapeutics have unwanted side effects, especially at the doses needed for pharmacological treatment.

Different mechanisms, such as glutamate-induced excitotoxicity, oxidative damage, excess production of nitric oxide, and damage to cell membranes by free radicals and peroxynitrite that induce lipid peroxidation are largely responsible for the formation of secondary injury [30]. Although the etiology and pathogenesis of spinal cord injury are not fully understood, previous studies have suggested that ROS and oxidative stress play an important role in the physiopathology of that injury [31]. The presence of high concentrations of ROS and RNS in the injured area after trauma damages cell proteins, lipids, and nucleic acids, contribute to the progression of secondary injury [32]. Therefore, reducing oxidative stress may be an effective strategy for therapeutic intervention in these injuries.

Under normal conditions, there is a dynamic balance (i.e., homeostasis) between the potential for oxidative damage and the natural antioxidant defense system. Following spinal cord injury, macrophages and microglial cells produce excessive amounts of nitric oxide, superoxide anions, H_2_O_2_, and ^•^OH in the extracellular space. This leads to the deterioration of that dynamic balance [9,11,33]. 

In a spinal cord injury, activated astrocytes and microglial cells migrate into the lesion. T cells are required for macrophage activation and cellular immune response [34]. Macrophages and microglia are also involved in the secondary pathological and inflammatory response by releasing pro-inflammatory cytokines such as TNF-α, IL-1, and IL-6 [35]. Indeed, studies have also shown an increase in TNF-α and IL-6 levels in these types of injuries [36]. The increased pro-inflammatory cytokines further induce upregulations in ROS and RNS production, and these oxidants in turn promote further inflammatory cytokine release. This results in a cyclic cytotoxic crosstalk contributing to damage and activation of other cellular processes. For example, a large amount of chondroitin sulfate, a proteoglycan that suppresses the growth of axons, begins to be expressed in astrocytes, which contributes to the formation of glial scar tissue [37]. Gliosis is a common parenchymal reaction in the central nervous system and an indicator of a pathological process. It is considered a major physical and chemical barrier to axonal regeneration [38,39]. Leukocyte infiltration exacerbates demyelination of the axons beginning especially within the first 24 h and peaking over the subsequent days [40]. Early initiation of treatment is crucial for reducing gliosis and demyelination. Therefore, in the present study, we began intraperitoneally (i.p.) treatment with hydrogen-rich saline on the first day after spinal cord injury and continued this treatment for seven days.

IL-6 is a multifunctional cytokine with immunological and metabolic roles. It is generally considered to be a nonspecific manifestation of inflammation that is released in response to infection, burns, trauma, and neoplasia [41]. Measuring the IL-6 level in the blood is useful for detecting the inflammatory state [42], and it has been revealed that IL-6 also plays a role in the differentiation and proliferation of fibroblasts, osteoclasts, hematopoietic stem cells, and neural stem cells [43]. IL-6 release has been shown to be important in many diseases, such as Crohn’s disease and rheumatoid arthritis [44], and studies have suggested that it also plays an important role in inflammatory reactions in spinal cord injury and acts as a neurotoxic agent [45]. Some studies have indicated that suppression of IL-6 production during the acute stage of spinal cord injury reduces the formation of gliosis [46]. In our study, we showed that IL-6 levels increased after trauma and that IL-6 levels were significantly decreased in the group treated with hydrogen-rich saline, which was administered i.p.. This finding that HRS can decrease IL-6 levels is corroborated by other studies [13].

TNF-α, a cell-signaling protein involved in systemic inflammation, is one of the cytokines that fuel the acute phase reaction. This protein plays a role in the adhesion of neutrophils to endothelial cells and causes the production of some cytokines [47].

In general, apoptosis is a programmed event during which the cell activates a number of metabolic and physiological processes that result in self-destruction. It is regulated by genes, requires RNA, protein synthesis, and ATP, and it maintains homeostasis within the organism [48]. Apoptosis is known to be triggered by the release of cytokines, inflammatory damage, free radical injury, and excitotoxicity after traumatic spinal cord injury [49]. It has also been suggested that apoptosis in spinal cord injury negatively affects the results by increasing neuronal loss [50]. Receptor-dependent apoptosis is stimulated by extracellular signals, especially TNF-α [51], which is a pleiotropic proinflammatory cytokine that rapidly accumulates within the damaged area after spinal cord injury and causes apoptosis using receptors on the cell surface [52]. TNF-α also acts as an external signal that triggers apoptosis in neurons and oligodendrocytes after the injury [53]. This apoptosis peaks approximately 24–48 h after injury [54]. The present study showed that, as expected, TNF-α levels increased after spinal trauma. However, TNF-α levels significantly decreased in the group that was i.p. administered a hydrogen-rich saline. A decrease in excessive levels of TNF-α may subsequently decrease apoptosis. Many studies have previously demonstrated that hydrogen therapy can decrease apoptosis and levels of TNF-α [13].

Importantly, there have been only a few experimental models of spinal cord injury with hydrogen-rich saline. For example, Chen et al. (2010) demonstrated that after experimental spinal cord injury in rats, administration of hydrogen-rich saline decreased markers of neuronal damage and apoptosis such as caspase-3, caspase-12, and TUNEL-positive cells [9]. HRS administration also reduced oxidative stress as evidenced by decreased malonaldehyde (MDA), an end-product of lipid peroxidation, and myeloperoxidase (MPO) activity. MPO is expressed by neutrophils and generates ROS that contributes to the secondary injury. They also reported that HRS increased brain-derived neurotrophic factor (BDNF), which plays a major role in neuronal survival. Lastly, they also observed an improved locomotor function and concluded that HRS protected spinal cord contusion injury [9]. 

Another study used the inhalation of various concentrations of molecular hydrogen 10 min before and for 60 min after spinal cord injury induced by ischemia-reperfusion in rabbits [55]. In corroboration with our study, they also found a decrease in inflammation and other markers. They reported that hydrogen inhalation decreased levels of inflammation, (e.g., TNF-α and high-mobility group box 1), oxidative stress (e.g., 8-iso-prostglandin F2 α, and MDA), neuronal apoptosis (e.g., (caspase-3 activity and number of TUNEL positive neurons), and increased markers of antioxidant enzymes (e.g., superoxide dismutase, catalase) in the serum and spinal cord. Phenotypically, they also found that H_2_ treatment prevented the significant decrease in number of normal motor neurons and ameliorated hind-limb motor dysfunction. Importantly, only a 2% and 4% H_2_ concentration were effective and not a 1% H_2_ concentration. In our study of injecting HRS, the concentration of H_2_ in the plasma is expected to reach approximately ≈11 µM. This is close to the expected plasma concentration following inhalation of 2% H_2_ gas predicted by Henry’s Law (≈14 µM). However, the differences may also be important: the concentration of HRS would decrease to baseline within ≈60 min, whereas inhalation would remain at that concentration for as long as the gas is being inhaled, in this case, a total of 70 min. However, in contrast to their study, we administered hydrogen daily for seven days. More research needs to be conducted to determine the best method of administration, as well as to optimize the dose, frequency, and duration of H_2_ therapy. This may also include changing partial pressures (concentrations) of oxygen gas, which can create meaningful biphasic responses in ROS production and inflammatory signaling [56].

Several other studies also investigated the effects of molecular hydrogen on spinal cord injury. For example, Chen X. et al. [57] incubated mechanically injured spinal cord neurons with hydrogen-infused media in vitro and demonstrated that hydrogen has protective properties in spinal cord neurons due to its anti-apoptotic properties. Similarly, Jian-long Wang et al. [58] infused hydrogen-rich saline into the subarachnoid space for therapeutic purposes in experimentally induced spinal cord injury in rats, and reported that hydrogen attenuates spinal cord damage through a mechanism involving the antioxidant system, calcitonin gene-related peptide, and caspase-3. In the present study, we found results similar to those in the literature, which showed that apoptotic cells were reduced with hydrogen-rich saline treatment in spinal cord injury.

It has been observed that IL-6 and TNF-α levels are greatly increased in the acute stages of experimental spinal cord injury in rats [59,60]. In this study, similar to what is reported in the literature, IL-6 and TNF-α levels increased in rats following spinal cord damage but were significantly decreased following HRS administration. Taken together, these results in conjunction with other studies on molecular hydrogen, suggest that HRS therapy may be clinically useful for spinal cord injuries. However, further investigation is needed to determine the exact pharmacokinetics, the molecular mechanisms, the optimal method of administration (e.g., HRS or inhalation of H_2_), as well as the dose and frequency of hydrogen therapy.

## 4. Materials and Methods

### 4.1. Ethics and Study Design

This research started after getting approval from Kafkas University Animal Experiments Local Ethics Committee Kars (Code No:2021/030, Date: 25 March 2021). The study was carried out at Kafkas University Experimental Animals Application and Research Center. Twenty-four four-month-old male Sprague Dawley rats, each weighing approximately 225–250 g, were housed at appropriate room temperature and allowed ad libitum access to water and feed. The rats were divided into four groups with six rats in each group as follows:

1. Control (C): laminectomy only.
2. Spinal cord injury (SI): after laminectomy at T7–T10, the dura was left intact and the Tator and Rivlin clip compression model was applied to the spinal cord for 1 min; no other treatment was applied.
3. Hydrogen (H): laminectomy at the T7–T10 level followed by treatment with i.p. hydrogen-rich saline for 7 days.
4. Spinal cord injury-hydrogen (SI-H): hydrogen-rich saline administered i.p. for 7 days after laminectomy at T7–T10, leaving the dura intact and applying the Tator and Rivlin clip compression model to the spinal cord for 1 minute.

### 4.2. Surgical Procedure

Before surgical applications, each experimental animal was provided with an intramuscular 10 mg/kg xylazine (5 mg/kg; BioVeta, Ankara, Türkiye) and 80 mg/kg ketamine HCl (Pfizer, Istanbul, Türkiye). After the back area was shaved, it was sterilized with povidone iodine. The middle line skin incision was made between T5 and T12. The lumbosacral fascia was opened as longitudinal and the paraspinal muscles were disrupted bilateral subperiosteal to reveal the T7–T10 lamina. After the T7–T10 laminectomy, epidural adipose tissue was removed and the dura mater was released. During these procedures, a dural defect was not observed. Hemostasis was achieved with Bipolar Cotter. The epidural cavity was expanded laterally to fit the standard spinal trauma model (Rivlin and Tator; Spinal Cord Trauma Model) [61]. Group 2, 3 rats were damaged at T9 level for one minute using the aneurysm clip (Yasargil Fe 750, Valley, Aesculap, PA, USA). The post-trauma layers were closed in the anatomical plane and the surgical procedure was terminated by ensuring that the rats woke up normally. At any stage of the surgical procedure, rats whose dura was damaged were excluded from the study. After day 7, a lethal dose of Pentobarbital (200 mg/kg, Biorta, Ankara, Türkiye) was administered to the rats. Serum was taken from the intracardium of the rats. The surgery area was then reopened and the damaged spinal cord was carefully removed. Samples of the spinal cord were evaluated histopathologically.

### 4.3. Hydrogen-Rich Saline Procedure

Hydrogen-rich saline was prepared in the Innovative Food Technologies Development, Application, and Research Center, Iğdır University (Türkiye) according to the method of Bulut et al. [62]. Briefly, a thick glass tube was filled with of 0.9% NaCl sterile solution instead of pure water. Hydrogen gas was then dissolved into the saline solution for 6 h under high pressure (0.4 MPa). This method has been shown to make a hydrogen concentration of 0.65 to 0.85 mM, as determined via gas chromatography [63]. As we did not have access to gas chromatography, we confirmed that the saline was saturated with hydrogen by using the ORP electrode (Sensorex, Garden Grow, CA, USA). The ORP method, although not the gold standard [64], can be used to ensure that saturation of H_2_ is achieved by continuous bubbling of H_2_ until the ORP reading does not further decrease. Hydrogen-rich water has a half-life of approximately two hours [64]. HRS tubes without headspace were prepared daily for 7 days followed by storage at 4 °C until use, which at most was 4 h later.

### 4.4. Treatment

After the experimental spinal cord injury, the treatment of i.p. HRS (10 mL/kg) was applied on the first day and repeated daily at the same time for seven days. Based on the volume and concentration, the dose of H_2_ administered is estimated to be approximately 1.8 micromoles, which when dissolved in the fluid of the rats (assuming 70% water) would reach a concentration of ≈11 µM

### 4.5. Histological Analyses

After being immersed in 10% formaldehyde for 72 h, the tissue from all groups was stained for histopathological examination. The samples were washed in running water for 4 h and then dehydrated by passing them through a series of increasing degrees of alcohol. After the tissues were dehydrated, they were cut into sections and cleared, infiltration was performed by passing through the paraffin series, and the tissues were embedded in paraffin blocks. The tissues were then stained using H & E and terminal TUNEL.

For TUNEL staining, five fields were randomly selected from each slide and the number of positive cells in these fields was counted. Five slides were evaluated in each group. TUNEL staining was used to detect cellular apoptosis in the spinal cord tissues from all groups according to the manufacturer’s protocol (Elebscience, Houston, TX, USA) TUNEL apoptosis assay kit [HRP-DAP] Cat no: E-CK-A331). After examining the TUNEL-positive cells in the spinal cord tissues using an Olympus BX43 microscope (Evident Corporation Tokyo, Japan), their photographs were taken using an Olympus DP21 camera (Evident Corporation Tokyo, Japan). The apoptotic cells showed intense dark nuclear staining. Apoptotic ratio = the number of TUNEL-positive cells/the total number of cells. 

### 4.6. Biochemical Measurements

The obtained serums were kept at −20 °C until analysis. TNF- α and IL-6 levels were determined in the intracardiac serum by using commercially available Enzyme-Linked Immunosorbent Assay (ELISA) (Elabscience, Houston, TX, USA) kits in compliance with the manufacturer’s instructions.

### 4.7. Statistical Analyses

Before the study, the sample size was determined by power analysis using G-Power version 3.1.9.7 (Heinrich Heine University Düsseldorf, Germany). The sample size was calculated according to the test power of 0.96 and significance level of 0.05 according to the G-Power test. ANOVA (Tukey) analysis was performed for TNF- α and IL-6 parameters. A *p* value of <0.05 was considered significant in the analyses. GraphPad version 8.1.0 (GraphPad Software, San Diego, CA, USA) was used for statistical analysis. The Tukey’s test of one-way analysis of variance was conducted using SPSS version 22 (IBM Corp., Armonk, NY, USA) to count the alfa -positive cells among the groups.

## 5. Conclusions

The results of the present study demonstrate that, after experimental spinal cord injury in rats, hydrogen-rich saline treatment significantly decreased inflammation as evidenced by decreased IL-6 and TNF-α levels. Consequently, this resulted in histopathologically reduced TUNEL-positive apoptotic cells. Taken together, hydrogen-rich saline therapy after spinal cord injury reduces inflammatory cytokines and apoptosis, which may qualify it as a potentially effective therapeutic agent for treating the condition. Specifically, the results of this study may have relevance for patients with trauma-induced spinal cord injury, as the treatment protocol may consist of hydrogen alone or in combination with other anti-apoptotic agents. However, prospective, multicenter, randomized, double-blinded clinical studies are necessary to confirm our results and determine the clinical effectiveness of hydrogen-rich saline therapy.

## Data Availability

Data is contained within the article.
